# Global health education programs: Are we embedding contemporary global health needs into the curriculum of master’s programs?

**DOI:** 10.3389/fpubh.2025.1697295

**Published:** 2026-01-09

**Authors:** Samraj Singh Bhullar, Antonia Roberts, Báltica Cabieses, Edward Mezones-Holguín, Ali Al-kassab-Cordova, Manuel Espinoza

**Affiliations:** 1 Hull York Medical School, University of York, York, United Kingdom; 2 Centro de Salud Global Intercultural (CeSGI), Facultad de Medicina Clínica Alemana, Facultad de Psicología, Universidad del Desarrollo, Santiago, Chile; 3 Department of Health Sciences, University of York, York, United Kingdom; 4 Center of Excellence in Economic and Social Research in Health, Universidad San Ignacio de Loyola, Lima, Peru; 5 Epi-gnosis Solutions, Piura, Peru; 6 School of Public Health, LKS Faculty of Medicine, The University of Hong Kong, Hong Kong SAR, China

**Keywords:** global health, global health education, global north, global south, equity

## Abstract

**Introduction:**

Global health education (GHE) is expected to prepare professionals to address complex, interlinked global challenges. However, current GHE structures often reflect persistent power asymmetries between the Global North and South, limiting the development of a truly global and equitable health workforce. This review examines how global health master’s programs are distributed geographically and to what extent their thematic focus and core curricular content reflect current global health priorities, particularly those related to equity and social justice.

**Methods:**

A mapping review of 86 graduate-level GHE programs worldwide was conducted to examine their geographic distribution, thematic focus, and curricular content. Programs were categorized by region and analyzed for thematic emphasis and pedagogical approaches, based on publicly available information on modules and learning activities.

**Results:**

The review found that 84% of GHE programs are offered by institutions in the Global North. Programs in the Global South are fewer but tend to emphasize environmental health, governance, and community engagement, often incorporating experiential learning. Across all regions, key topics such as health systems, global health challenges, sustainability, law, ethics, and human rights are unevenly integrated. This variability risks producing graduates with inconsistent competencies to address global health priorities. The dominance of Global North institutions in GHE reflects broader structural inequities in global health. While emerging North–South and South–South collaborations and field-based learning suggest a shift toward more reciprocal models, many programs lack clearly defined aims and accountability frameworks.

**Discussion:**

To advance GHE, curricula must embed equity, interdisciplinarity, and regional relevance. Explicit learning outcomes should include power analysis and partnership-building, co-designed and co-delivered with institutions and communities from both the Global North and South. Such reforms are essential to cultivate a workforce capable of addressing global health challenges with contextual sensitivity and systemic insight.

## 1 Introduction

Over the past decade, a convergence of global crises, from pandemic threats to the escalating burden of chronic diseases, has made it increasingly clear that safeguarding population health can no longer be achieved through isolated national strategies (1, 2). These challenges transcend borders, demanding coordinated, cross-sectoral responses. In recognition of this interdependence, the Lancet Commission on the education of health professionals for the 21st century called for a transformative shift in training paradigms. It emphasized the need to prepare future health professionals to operate across disciplines, cultures, and jurisdictions, and to translate global solidarity into meaningful local action (3). This vision aligns with international health and development agendas, including the Sustainable Development Goals, which call for reductions in premature mortality and improvements in quality of life worldwide. As such, reimagining health education is not only a pedagogical imperative but also a strategic necessity for advancing equitable and sustainable health outcomes globally (4).

Despite notable progress in elevating global health as a distinct field, debates over its definition remain both persistent and consequential (5, 6). One of the earliest conceptualizations described global health as addressing health issues that transcend national boundaries and require action on global determinants of health (7). While groundbreaking at the time, this definition was critiqued for its lack of specificity, passive framing, and omission of collaboration and research as central pillars (8). A more widely adopted definition by Koplan et al. (9) reframes global health as a domain of study, research, and practice aimed at improving health and achieving equity for all people worldwide. This approach emphasizes transnational health challenges, interdisciplinary collaboration beyond the health sciences, and the integration of population-level prevention with individual care. These evolving definitions reflect a growing recognition of the complexity and interconnectedness of global health, aligning closely with the 2030 Agenda for Sustainable Development. In particular, Sustainable Development Goal 3—“Ensure healthy lives and promote well-being for all at all ages”—embodies the equity-driven ethos of contemporary global health. It positions health not merely as an outcome but as a foundational condition for social and economic development, while also calling attention to the structural power imbalances that shape health outcomes globally (10).

In the evolving landscape of global health education (GHE), efforts to define and standardize the field have gained momentum. The Consortium of Universities for Global Health (CUGH), established in 2008 as a membership-based organization, has played a pivotal role in shaping the discipline. It has sought to articulate a coherent definition of global health, harmonize curricula and competencies, and establish criteria for student and faculty exchanges (11). Notably, CUGH and other key actors, including governments and universities across diverse regions, have adopted the widely cited definition by Koplan et al., which frames global health as a domain of study, research, and practice committed to improving health and achieving equity worldwide (12–14). Recent empirical studies, such as the mixed-methods adaptation of the CUGH competency framework in China, illustrate how institutions are recalibrating their curricula around a shared set of global health competencies. This trend signals a growing recognition of the strategic importance of aligning health professional education with a genuinely global practice agenda (14). Further advancing this agenda, a coalition of global health experts has proposed a comprehensive roadmap that includes a unified definition of global health, sustainable funding mechanisms, measurable benchmarks, and a collaborative governance model designed to foster multilevel stakeholder engagement (15). These developments reflect a broader shift toward more integrated, equitable, and context-responsive approaches to global health training.

While certain foundational elements of GHE have gained consensus, the field remains dynamic, with ongoing debates about its core characteristics and conceptual boundaries. A systematic review of the literature (5) identified widespread critique of Koplan’s influential definition and documented 34 distinct original definitions of global health (5). From this diversity, the review distilled eight recurring dimensions that collectively characterize the field: (i) a domain encompassing research, healthcare, and education; (ii) inherently multifaceted-spanning disciplinary, sectoral, cultural, and national contexts; (iii) grounded in a normative commitment to equity; (iv) shaped by political dynamics and power relations; (v) problem-oriented in its approach; (vi) transcending national borders; (vii) influenced by globalization and international interdependence; and (viii) persistently perceived as definitionally ambiguous. These dimensions reflect both the richness and complexity of global health as a field in flux, one that resists reduction to a single framework and instead demands ongoing critical engagement with its evolving scope and purpose.

Despite growing consensus on the importance of global health education (GHE), critical scholarship continues to interrogate its historical foundations and persistent inequities. Much of the critique centers on the field’s enduring ties to tropical medicine and international health—domains historically shaped by colonial and Global North perspectives that often marginalize diverse voices and experiences (16, 17). In this *Review,* the terms “Global North” and “Global South” are used as heuristic geopolitical categories rather than strictly geographic ones. “Global North” broadly refers to high-income countries and historically dominant centers of political and economic power, whereas “Global South” denotes countries and regions that have been structurally disadvantaged within the global economic and political order, many of which are classified as low- and middle-income countries (18).

International health traditionally focused on disease and health needs in low- and middle-income countries (LMICs), building on the legacy of tropical medicine (19), which emerged during colonial expansion as settlers encountered unfamiliar disease ecologies in the tropics (20, 21). These origins have left a lasting imprint on global health, contributing to ongoing tensions between the Global North and South in defining, practicing, and teaching the discipline. For this Review is important to state that the terms “low- and middle-income countries” ($1,135–$13,935), (LMICs) and “high-income countries” ($13,935 or more) (HICs) are classified following the World Bank income 2026 classification, which is based on gross national income per capita (22).

Although global health aspires to equity, much of its scholarly production and institutional leadership remains concentrated in the Global North. This imbalance reinforces a North-assists-South paradigm, positioning Northern institutions as the primary generators of knowledge and Southern actors as recipients, thereby perpetuating colonial structural asymmetries in the field. Even within the Global North, definitions of global health and curricular approaches vary widely, resulting in a lack of standardization across programs. Nonetheless, GHE is increasingly offered at multiple levels, though access remains uneven. Most programs are located in high-income countries (HICs) or are prohibitively expensive for students in LMICs, limiting participation and leadership from the Global South (23). Moreover, donor-driven agendas often steer graduates away from Southern priorities, while scholars from LMICs frequently depend on Northern institutions for resources, recognition, and career advancement (20, 24). These dynamics underscore the urgent need to decolonize global health education and foster more equitable, reciprocal partnerships that reflect the diverse realities and priorities of global communities.

Despite persistent tensions and structural inequities, GHE has expanded rapidly over the past decade, with programs now offered across a wide spectrum, from undergraduate to postdoctoral levels (25). However, this proliferation has largely unfolded without coordinated oversight, resulting in a fragmented landscape of competencies and curricular standards. Efforts to define core GHE competencies have been complicated by the field’s inherently interdisciplinary nature and its diverse geographical contexts (26). In this environment, a reconceptualization of global health is increasingly urgent to safeguard the discipline’s coherence, relevance, and transformative potential (27). In this *Review,* we understand “current global health priorities” as those that center equity, address structural power imbalances, and respond to decolonial critiques of the field. Therefore, this study addresses the following research question: *How are global health master’s programs worldwide distributed geographically, and to what extent do their thematic focus and core curricular contents reflect current global health priorities?*

In this *Review*, we argue that the current profusion of programs, each shaped by distinct epistemic traditions, competency frameworks, and geopolitical incentives, not only reflects but often amplifies the very power imbalances that global health seeks to dismantle. By mapping where programs are located and what they teach, we seek to make these dynamics visible and to identify concrete entry points for more equity and justice-oriented reforms in GHE. Drawing on emerging mapping evidence and a comparative analysis of curricular offerings across regions and income settings, we identify areas of convergence, persistent blind spots, and promising pedagogical innovations. Rather than prescribing a singular model, we aim to illuminate patterns that can guide universities, professional societies, and funders in recalibrating educational agendas. By fostering more equitable, context-responsive approaches to training, global health education can better prepare practitioners to engage with the complexities of health across borders and systems.

To investigate the current landscape of global health education (GHE), we conducted an exploratory mapping review of graduate-level master’s programs across continents, deliberately excluding PhD programs due to their distinct academic orientation. This methodological approach enabled the identification of existing educational offerings and facilitated the analysis of emerging trends and interregional linkages (28). The review was informed by similar studies that have previously examined the structure and distribution of GHE programs globally (29). In January 2024, we compiled a list of the top 25 to 50 universities per continent using standardized metrics from the QS World University Rankings (30). For countries not represented in the QS rankings, supplementary online research was conducted to identify nationally recognized institutions. Each university’s website was systematically searched to determine the presence of graduate-level global health programs. Rankings may have varied between the time of data collection and the present. To enhance coverage, targeted keyword searches were performed using Google, incorporating terms such as “global health,” “graduate,” and “postgraduate,” translated as appropriate for each country. Additional institutions identified during the literature review were also included. In total, 106 programs were initially identified. The flowchart of the programs selection process is available in Supplementary material 1. Of these, 20 were excluded: 18 due to insufficient publicly available data and 2 for lacking a specific focus on global health, resulting in a final sample of 86 graduate programs (Table 1). For each included program, publicly accessible information was analyzed, focusing on (i) the geographic location of the institution and (ii) the titles of individual modules and description and objectives when they were available. Due to limited access to detailed syllabi, most modules were grouped and analyzed based on their explicit titles, allowing for a comparative overview of curricular content across diverse educational settings.

**Table 1:** Summary of 86 GHE programs from selected top 25–50 universities by continent.

Country	Institute	Title
Australia	University of New South Wales (UNSW Sydney)	Master of Global Health
	University of New South Wales (UNSW Sydney)	Master of Global Health (Extension)
	University of New South Wales (UNSW Sydney)	Master of Global Health/Master of Health Leadership and Management
	University of New South Wales (UNSW Sydney)	Master of Global Health/Master of Infectious Diseases Intelligence (Extension)
	University of New South Wales (UNSW Sydney)	Master of Global Health/Master of Public Health (Extension)
	Victoria University Melbourne	Master of Global Public Health
Canada	McMaster University	Master of Science in Global Health
	University of Alberta	MSc Global Health
	University of Ottawa	Master’s in Public Health, Global Health Stream
Chile	Pontifical Catholic University of Chile (UC)	Magíster en Salud Pública Global
China	Duke Kunshan University	MSc Global Health
	Tsinghua University	International Master of Public Health (IMPH)
Denmark	University of Copenhagen	Master of Science (MSc) in Global Health
Egypt	The American University in Cairo	MPH Global Public Health
Germany	Deggendorf Institute of Technology	MSc Global Public Health
Italy	University of Milan	Master in Global Health
Japan	Tokyo Medical and Dental University	MPH of Public Health in Global Health
Kenya	Kenya Medical Research Institute	MSc Global Health
Nepal	Kathmandu University	MSc Public Health in Global Health
Netherlands	Maastricht University	Masters Global Health
	Vrije Universiteit Amsterdam	Research Master’s in Global Health
Nigeria	Bayero University	MSc Global Health and Politics
Norway	University of Bergen	Master’s Program in Global Health
Peru	Cayetano Heredia Peruvian University	Maestría en Salud Pública y Salud Global
Rwanda	University of Global Health Equity	Master of Science in Global Health Delivery
Spain	Universitat de Barcelona - IS Global	Master of Global Health
Sweden	Karolinska Institut	MSc global health
	Uppsala University	Master of Medical Science with Global Health
Taiwan	National Taiwan University (NTU)	Masters Global Health
	Taipei Medical University	Master Program in Global Health and Health Security
Thailand	Thammasat University	MPH of Public Health in Global Health
Turkey	Koç Üniversitesi	MSc Global Health
United Kingdom	Brighton and Sussex Medical School	MSc Global Health
	Canterbury Christ Church University	MSc Global Public Health
	De Montfort University	MSc Global Health
	Imperial College London	MPH Global Master of Public Health
	King’s College London	MSc Global Health
	King’s College London	MSc Global Health, Social Justice, Public Policy
	Liverpool School of Tropical Medicine	MSc Global Health
	London School of Hygiene and Tropical Medicine, University of London	MSc Global Health Policy
	Manchester Metropolitan University	MPH Global Public Health
	Newcastle University	MSc Global Public Health
	Oxford Brookes University	MPH Global Public Health Leadership
	Queen Margaret University	MSc Global Health
	Queen Margaret University	MSc Applied Global Health
	Queen Mary University of London	MSc Global Public Health (Online)
	Queen Mary University of London	MSc Global Public Health and Policy
	Queen Mary University of London	MRes Global Public Health and Policy
	Queen Mary University of London	MA Development and Global Health
	Queen’s University Belfast	MPH Global Health
	The University of Manchester	MSc Global Health
	The University of Manchester	MPH Master of Public Health (Global Health)
	The University of Nottingham	MPH Public Health (Global Health)
	The University of Winchester	MSc Global Health
	University College London	MSc Global Health and Development
	University of Aberdeen	MSc Global Health and Management
	University of Bath	MSc Global Public Health and Policy
	University of Essex	MSc Global Public Health
	University of Glasgow	MSc Global Health
	University of Birmingham	MPH Public Health (Global Health)
	University of Greenwich	MSc Global Public Health
	University of Huddersfield	Master of Public Health (Global)
	University of Oxford	MSc Global Health Science and Epidemiology
	University of Plymouth	MSc Global Health
	University of Southampton	MSc Global Health
	University of Westminster	MSc Global Public Health with Data Science
United States	Arizona State University	MA Global Health
	Brown University	Master’s Global Public Health
	Duke University	MS Global Health
	Emory University	MPH in Global Environmental Health
	Emory University	MPH in Global Health
	George Mason University	MS Global Health
	Georgetown University	MS Global Health
	Harvard University	Master of Medical Sciences in Global Health Delivery
	Icahn School of Medicine at Mount Sinai	MPH of Public Health with speciality Global Health track
	Loma Linda University	MPH in Global Health
	Medical College of Wisconsin	MS Global Health Equity
	Michigan State University	MS Global Health
	Northwestern University	MS Global Health
	Ohio University	Master of Global Health
	Rutgers, The State University of New Jersey	MPH in Global Public Health
	Tufts University	MS in Infectious Diseases and Global Health
	University of California, San Francisco	MS Global Health
	University of Maryland	MS Global Health
	University of Miami	M. A. in Global Health and Society
	University of Notre Dame	MS Global Health

The mapping process involved systematically categorizing the mandatory modules offered by identified graduate global health programs according to a predefined list and thematic descriptions of global health content. This list was created *a priori* through a literature review of global health competencies, enabling the identification of competencies relevant to the discipline from diverse perspectives. The competencies identified in the literature are presented in Supplementary material 2. During coding, each module was classified into the category that best represented its focus. Only the categories with modules are presented in the results of this *Review*. To ensure accuracy, the categorization was conducted in two iterative rounds by one researcher. To enhance consistency and reduce subjective bias, coding decisions and ambiguous cases were regularly reviewed and discussed with two other authors. The final thematic framework was agreed upon by the research team. Data from the mapping exercise were recorded in Excel spreadsheets and subsequently analyzed using built-in formulas and visualized through charts. This facilitated the identification of curricular patterns and thematic distributions across programs. In parallel, we documented distinctive features and notable characteristics of individual programs, which provided additional insights into the pedagogical diversity and innovation present within global health education globally.

## 2 Global health education in current times: disparities between Global North and Global South

**Figure 1:**
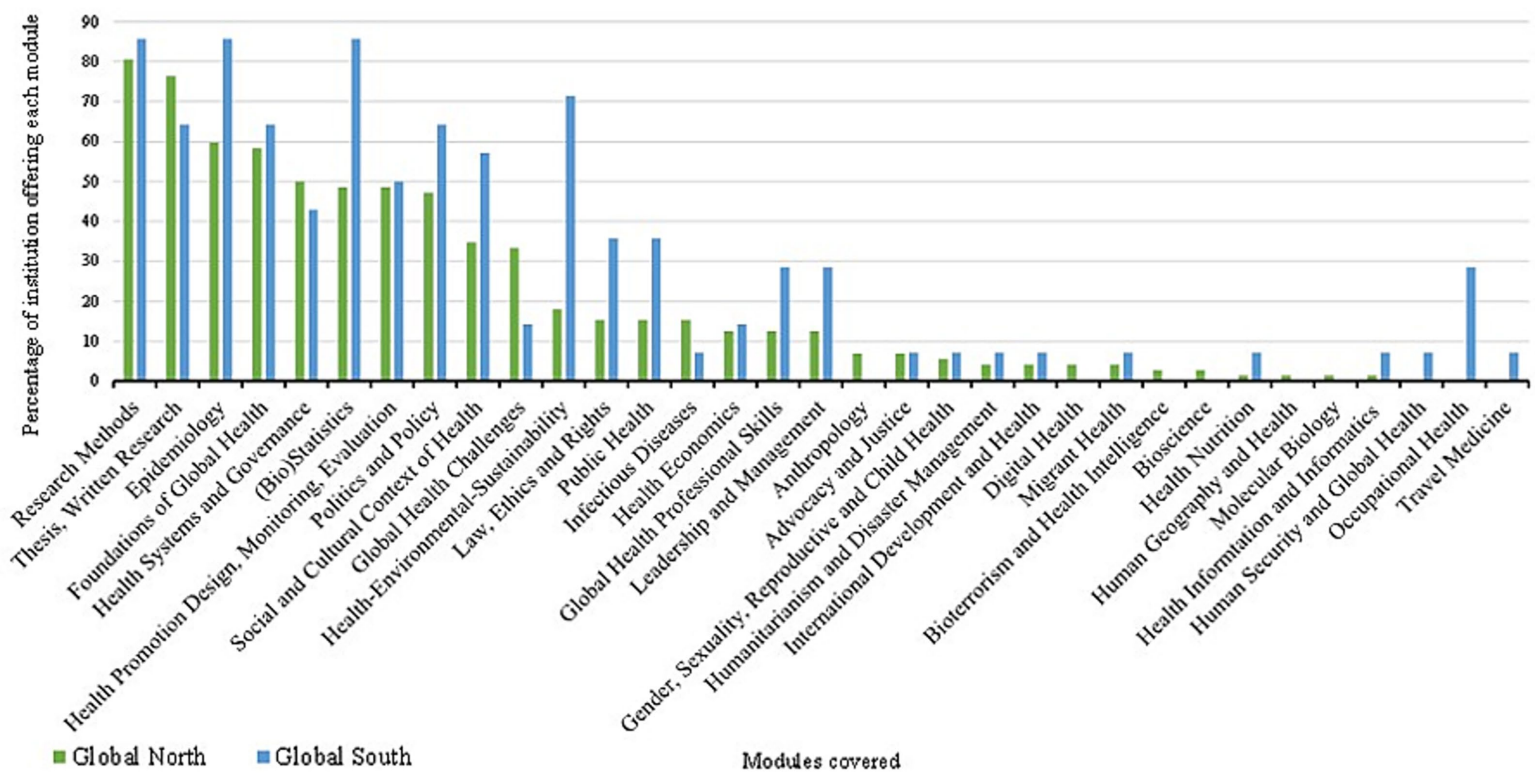
Bar chart of graduate programmes modules coverage in Global North and Global South. Bars in green represent Global North Programmes, and blue bars represent Global South Programmes.

The majority of graduate global health programs identified in this review are concentrated in the Global North, with 72 programs (84%) located in high-income countries, compared to only 14 programs (16%) in the Global South. Within the Global North, Europe accounts for the largest share (*n* = 43), with the United Kingdom emerging as the most prominent contributor (*n* = 34). North America follows with 23 programs, predominantly in the United States (*n* = 20) and Canada (*n* = 3), while Australia hosts six programs. In contrast, the Global South presents a more limited distribution: Asia offers eight programs, primarily in China and Taiwan; Africa accounts for four; and South America for two. A detailed visual representation of this distribution is provided in Supplementary material 3. A total of 33 distinct modules were identified across the graduate global health programs included in this review. Among these, a core set of modules emerged as consistently taught across international institutions. These include research methods, thesis-based research, epidemiology, foundations of global health, biostatistics, health promotion design, monitoring and evaluation, health politics and policy, and the social and cultural contexts of health. These foundational components appear to constitute the pedagogical backbone of global health education globally. In contrast, a second tier of modules was found to be more variably integrated across programs. These include health systems and governance, global health challenges, health-environment-sustainability intersections, law, ethics and human rights, public health, infectious diseases, health economics, and leadership and management. To assess thematic coverage across regions, we calculated the percentage of institutions in the Global North and Global South offering each module and visualized the results in Figure 1. To identify meaningful differences, we computed the percentage point gap in module coverage between Northern and Southern institutions. A variation of less than 10% was considered negligible, indicating comparable curricular emphasis across regions. Modules falling within this threshold included infectious diseases; health systems and governance; health nutrition; health information and informatics; foundations of global health; humanitarianism and disaster management; international development and health; migrant health; health promotion design, monitoring, and evaluation; health economics; advocacy and justice; and gender, sexuality, reproductive and child health. These findings suggest areas of curricular convergence that may reflect shared pedagogical priorities, despite broader disparities in program distribution and access (Figure 1). A variation of more than 10% in module coverage between institutions in the Global North and Global South was considered a meaningful indicator of curricular divergence. Notably, programs in the Global North more frequently included modules such as *global health challenges* and *thesis-based research*, which emerged as the most prominent areas of difference. Additionally, several modules were found exclusively within Northern institutions, including *bioscience*, *anthropology*, *bioterrorism and health intelligence*, *digital health*, *human geography and health*, and *molecular biology*, reflecting a tendency toward specialized and technologically oriented content. Conversely, certain modules were unique to institutions in the Global South, such as *human security and global health*, *occupational health*, and *travel medicine*, suggesting a more context-responsive orientation. Furthermore, a range of modules were more commonly taught in the Global South than in the North, including (in descending order of difference): *health-environmental-sustainability*, *(bio)statistics*, *epidemiology*, *social and cultural context of health*, *law, ethics and rights*, *public health*, *politics and policy*, *global health professional skills*, and *leadership and management*. These findings point to distinct pedagogical priorities across regions, shaped by local health challenges, institutional capacities, and geopolitical contexts. A detailed comparison of module coverage is presented in Supplementary material 4.

## 3 Global health curricular innovations and collaborations

Beyond curricular content, emerging findings reveal that global health education (GHE) programs are increasingly incorporating innovative pedagogical approaches, notably fieldwork experiences and collaborative partnerships. Fieldwork opportunities allow students to apply theoretical knowledge and practical skills in real-world settings, fostering experiential learning and contextual understanding. Among the programs reviewed, 22 institutions offered international fieldwork components, with a proportionally higher representation in the Global South (six programs, 43%) compared to the Global North (16 programs, 22%).

Cross-national collaborative partnerships in education have increasingly involved institutions from both the Global North and Global South engaging in co-design and co-delivery of academic programs. These collaborations encompass North–North, North–South, and, more recently, South–South initiatives. A notable example includes a consortium of seven countries representing both hemispheres, which jointly organizes a two-week international Global Health Symposium. This event convenes students and faculty from participating institutions to critically engage with global health issues through a series of expert-led sessions. Participants also present their thesis proposals, receive constructive feedback from peers and faculty, engage in interdisciplinary networking, and deepen their understanding of cultural diversity (31).

A compelling example of South–South collaboration between Latin America and the Middle East is an innovative 18-month educational program designed to immerse students in real-world challenges across diverse regions of the Global South. Developed over a five-year period, the curriculum was informed by an extensive literature review on global health and sustainable development, iterative feedback from global experts and students, and pilot testing of its core components. Central to this initiative is the belief that studying and living across multiple Southern contexts is essential for cultivating future leaders capable of driving transformative change. The program emphasizes experiential learning and community engagement, equipping students with practical competencies for impactful work in health and development. Its pedagogical framework is anchored in six foundational pillars: global health, sustainable development, social entrepreneurship, social justice, transformative learning, and South–South collaboration (32).

## 4 Discussion

This review set out to address how global health master’s programs are distributed geographically, and what thematic patterns and core modules characterize their curricula worldwide. Findings highlight three main contributions. First, contemporary GHE reveals distinct emphases between institutions in the Global North and Global South. As our research demonstrates, the majority of graduate-level global health programs are concentrated in the Global North, particularly in Europe and North America. This pronounced global disparity in the provision of GHE reflects enduring structural inequalities within the field. The dominance of institutions from the Global North perpetuates a model of knowledge production and dissemination that privileges Northern epistemologies and expertise, often at the expense of perspectives from the Global South. Such imbalances risk reinforcing the very inequities that global health seeks to address, underscoring the need for more inclusive and geographically representative educational frameworks.

Second, across these programs, we identified 16 core and adjunct modules that consistently appear in curricula worldwide. However, the variability in how these themes are taught reflects divergent pedagogical approaches, which may contribute to a fragmented and diluted understanding of global health, especially within Northern institutions. This fragmentation may stem from the limited critical reflection on the purpose and content of GHE curricula during program development (24), resulting in diluted understandings of global health and inconsistent training outcomes. In contrast, programs in the Global South demonstrated greater thematic coherence, suggesting a shared perspective on the relevance of specific modules. Notably, these programs placed stronger emphasis on topics directly aligned with pressing regional health threats, such as environmental sustainability and climate-related health challenges. This alignment may indicate a more context-responsive approach to curriculum design, rooted in the lived realities and priorities of Southern populations.

Third, our findings highlight how current imbalances in the distribution and content of GHE programs intersect with broader debates on decolonizing global health and advancing social justice. In particular, scholars have critiqued the persistence of colonial legacies in longstanding programs, many of which continue to frame global health through outdated lenses such as international or tropical medicine (26). This critique is linked to the observation that some programs were developed without sufficient critical reflection on the epistemological foundations and intended purpose of global health education (29). Encouragingly, emerging efforts are beginning to challenge these historical patterns. Increasingly, programs are aligning with contemporary definitions of global health and prioritizing collaborative fieldwork experiences that foster equitable partnerships between institutions in the Global North and South. These initiatives, such as international symposia and multi-country curricula, promote intercultural learning, reciprocal engagement, and capacity-building. Notably, South–South collaborations are gaining prominence, challenging traditional hierarchies and foregrounding the Global South’s leadership in shaping educational agendas.

These emerging partnerships between institutions in the Global North and South—as well as among Southern nations themselves—may reflect a growing recognition of the South’s vital contributions to the global health landscape. This trend resonates with scholars who argue that high-income countries (HICs), predominantly located in the Global North, bear a responsibility to facilitate equitable knowledge exchange by actively supporting capacity-building efforts in LMICs (33). A key strategy for strengthening LMIC capacities involves directing funding to local institutions, thereby enabling them to lead research, education, and training initiatives (34). However, realizing the full potential of these collaborations requires addressing persistent structural barriers. These include uneven infrastructure distribution and the absence of clearly defined career pathways for global health graduates in LMICs, factors that continue to hinder meaningful shifts in power dynamics between North and South (23, 29).

Our mapping showed that some programs are increasingly incorporating fieldwork experiences in the curricula. However, the literature highlights persistent ambiguities in how fieldwork is defined and implemented. These experiences range from small-scale community interventions to large transnational research projects, yet they are often vaguely described and inconsistently aligned with the priorities and needs of host communities in LMICs (35). Moreover, students frequently spend limited time with host institutions, which can result in minimal contributions and, in some cases, impose additional burdens on local staff (34, 36). A further critique is the tendency to situate fieldwork exclusively in LMICs, overlooking marginalized populations within HICs (37) and missing opportunities to engage with global health challenges in local contexts (38). Given these limitations, there is a pressing need to broaden the conceptualization of fieldwork in GHE. Clearer definitions, ethical frameworks, and reciprocal engagement strategies are essential to ensure that fieldwork contributes meaningfully to global health development and fosters equitable partnerships between institutions and communities. Addressing these challenges requires a reimagining of fieldwork practices, sustained investment in Southern institutions, and a commitment to ethical, reciprocal partnerships that empower all stakeholders in global health education and transform its colonial roots.

Additionally, the recent COVID-19 pandemic has exposed vulnerabilities in global health systems and underscored the urgent need to reform GHE. The pandemic has highlighted the possible limitations of existing graduate-level GHE programs, especially their fragmented pedagogical approaches and lack of contextual responsiveness to health crises in LMICs. COVID-19 has amplified the critiques on colonial legacies and the absence of critical reflection on the epistemological foundations on the field (39, 40), demonstrating that global health threats do not respect borders and that effective responses require inclusive, locally grounded, and globally coordinated education and practice. The pandemic has also illuminated the importance of equitable partnerships between institutions in the Global North and South, as well as the need for curricula that prepare health professionals to navigate complex, interdependent systems under conditions of uncertainty and inequality (41). Moving forward, GHE must embrace a transformative agenda, one that integrates pandemic preparedness, health equity, and collaborative fieldwork, to cultivate a generation of practitioners capable of responding to both acute crises and long-standing structural injustices.

Taken together, the geographic asymmetries, uneven thematic coverage, and limited representation of Southern institutions identified in this review point to the need for a profound transformation of GHE. Social justice must be a foundational principle in the reimagining of global health curricula. It requires not only addressing health inequities but also transforming the institutional and pedagogical structures that sustain them. This includes integrating critical perspectives on race, gender, and class; promoting community-engaged learning; and ensuring that educational programs are accessible and relevant to students from diverse backgrounds (20). Democratic participation in global health education, where stakeholders from both the Global North and South have equal voice in decision-making, is essential to building sustainable and context-sensitive health systems (34). Furthermore, cross-regional partnerships must move beyond symbolic inclusion to foster genuine reciprocity and long-term collaboration. This involves equitable funding mechanisms, shared authorship in research, and institutional capacity-building that empowers Southern institutions to lead in education, research, and policy development (15, 42). By embedding principles of democracy, social justice, and cross-regional solidarity into the fabric of global health education, the field can evolve into a more inclusive, responsive, and transformative discipline.

Justice-oriented global health education initiatives must address structural barriers to participation, including socioeconomic inequities, gender disparities, and limited institutional capacity in underrepresented regions. Strengthening local educational infrastructures and fostering long-term, reciprocal partnerships between institutions in the Global North and South are vital to building resilient health systems and empowering future leaders in global health. Ultimately, the transformation of GHE requires a shift from a fragmented and often inequitable model to one that is globally attuned, ethically grounded, and committed to social justice. By addressing the gaps between territories and embedding equity into the core of medical and health professional training, we can ensure that future practitioners are equipped to deliver care that is both effective and just, regardless of the setting in which they work. To make these implications more concrete, Table 2 summarizes the Illustrative curricular and policy implications of the findings suggested by this Review, organized by domain.

**Table 2:** Illustrative curricular and policy implications of findings.

Domain	Illustrative curricular and policy implications of findings
Geographic distribution and representation	Develop more inclusive, geographically representative frameworks that increase the visibility, participation, and leadership of institutions in the Global South within GHE.
Curricular content, equity and colonial legacies	Reorient GHE curricula toward inclusivity, equity, and regional relevance, integrating health equity, social determinants of health, systemic racism, and colonial legacies as core (not peripheral) content. These curricular changes should be articulated with cross-regional partnerships that move beyond symbolic inclusion toward genuine reciprocity, shared authorship, and institutional capacity-building, directing funding and infrastructure support to Southern institutions and embedding democratic participation in governance and curriculum design.
Social justice, critical perspectives and pedagogy	Incorporate critical perspectives on race, gender, and class; promote community-engaged, context-sensitive pedagogies; and ensure that programs are accessible and relevant to students from diverse and historically marginalized backgrounds.

It is important to acknowledge that this *Review* is based on a mapping methodology that presents several limitations, which must be critically considered when interpreting its findings. First, the initial search and data extraction were conducted by a single researcher and subsequently reviewed by two co-authors. While this process aimed to ensure consistency, it may have introduced selection bias or inadvertent omissions, particularly regarding programs offered outside dominant academic networks. Second, due to limited access to comprehensive module descriptions across institutions, the analysis relied primarily on module titles. This approach risks misrepresenting curricular content, as titles may not fully capture the scope or depth of the material covered. Third, the sampling strategy focused on top-ranked institutions within each continent, which may have disproportionately favored institutions in the Global North, given their frequent presence in international rankings. This introduces a structural bias that may obscure the contributions and innovations of institutions in the Global South. Fourth, the review exclusively examined master’s-level programs, thereby excluding undergraduate and doctoral-level global health education, which may offer distinct pedagogical approaches and institutional dynamics. Future research should adopt a more inclusive lens to capture the full spectrum of global health education. In addition, this review did not systematically examine the financial dimension of these programs, including tuition fees, living costs, or the availability of scholarships and other funding mechanisms. As cost is a key determinant of who can access global health education, the absence of a comparative analysis of program affordability limits our ability to assess the equity implications of the current GHE landscape. Future studies should therefore incorporate systematic data on program costs and funding structures to understand better how financial barriers shape global participation in global health training.

Fifth, the review considered only programs explicitly labeled as “global health,” potentially overlooking relevant curricula under titles such as “international health,” “health equity,” or “public health with a global focus.” Expanding the scope to include these programs would enrich the analysis and provide a more nuanced understanding of the field. Moreover, future studies should move beyond module mapping to examine the competencies embedded within global health curricula. This would allow for a deeper exploration of how educational outcomes differ across regions and how they reflect broader commitments to social justice, equity, and democratic participation in global health. In light of these limitations, future mapping reviews should adopt more robust and inclusive methodologies that foreground the voices and practices of institutions in the Global South. Doing so will contribute to a more equitable and representative understanding of global health education and support the development of cross-regional partnerships grounded in mutual respect and shared learning.

Based on the findings of our exploratory mapping review, we contend that GHE must undergo a profound transformation to adequately prepare medical and health professionals for the evolving and complex health challenges of the 21st century. The current structure of GHE, particularly within institutions in the Global North, often fails to reflect the realities of healthcare delivery in LMICs (45), where resource constraints, systemic inequities, and sociopolitical instability shape health outcomes. As such, there is an urgent need to reorient GHE curricula toward inclusivity, equity, and regional relevance. Medical and health professional training must be grounded in a framework that acknowledges and addresses the social determinants of health, systemic racism, and the historical legacies of colonialism that continue to influence global health practice (43). Integrating health equity content into core curricula, not as peripheral or elective topics but as foundational knowledge, is essential to cultivating culturally competent and socially accountable practitioners (43). Moreover, professionalism in healthcare must be redefined to include principles of equity, diversity, inclusion (EDI), cultural humility, and advocacy, particularly in training environments that serve historically marginalized populations (44).

Efforts to standardize medical education globally, such as those led by the World Health Organization (WHO) and the World Federation for Medical Education, have aimed to harmonize competencies across regions. However, these initiatives have often struggled to reconcile global standards with local needs, risking the imposition of Northern-centric models that may not be appropriate or effective in diverse healthcare settings and that might reproduce colonial legacies in the field (44). A more democratic and participatory approach to curriculum design, one that includes voices from LMICs and prioritizes bidirectional learning, is critical to ensuring that GHE is responsive to local contexts and emerging health threats.

A critical and reflexive approach to GHE, one that interrogates its epistemological foundations and embraces pluralistic, context-sensitive knowledge systems, can enhance professional competencies while simultaneously reshaping the field itself. Such an approach must centre equity, social justice, and democratic participation, fostering a generation of health professionals who are not only technically proficient but also ethically grounded and politically aware. By embedding these principles into the core of global health education, the field can move beyond rhetorical commitments to equity and begin to realize its transformative potential in both discourse and practice.
