# Supplementary material for: Global health education programs: Are we embedding contemporary global health needs into the curriculum of master’s programs?
Source: Front Public Health. 2026 Jan 9;13:1697295. doi: 10.3389/fpubh.2025.1697295 (PMC12827718; doi:10.3389/fpubh.2025.1697295)
Supplement: Supplementary file 1 [file Table_1.docx]

**Supplementary material 1. Inclusion-exclusion of graduate global health programmes for this study.**

Graduate GH master’s programmes identified from online

(n=89)

Graduate GH master’s programmes identified from literature

(n=17)

Graduate GH master’s programmes included in mapping study (n=86)

Degrees excluded for reasons that did not meet search criteria (n=20):

-No data available (n=18)

-Not global health (n=2)
