# Supplementary material for: Global health education programs: Are we embedding contemporary global health needs into the curriculum of master’s programs?
Source: Front Public Health. 2026 Jan 9;13:1697295. doi: 10.3389/fpubh.2025.1697295 (PMC12827718; doi:10.3389/fpubh.2025.1697295)
Supplement: Supplementary file 2 [file Table_2.docx]

**Supplementary material 2. Global health competencies found through the literature.**

List of competencies used for the analysis

| **Competencies** |
| --- |
| Global burden of disease |
| Globalization of health and healthcare |
| Social and environmental determinants of health |
| Capacity strengthening collaboration, partnering and communication |
| Ethics |
| Professional practice |
| Health equity and social justice |
| Programme management |
| Sociocultural and political awareness |
| Strategic analysis |
| Global health systems and international relations |
| Global evidence ecosystem |
| Role of international organizations |
| Universal health issues |
| Intellectual property rights |
| Responses to issues affecting different at-risk groups |
| Local, national, and international policy and economic context affecting global health |
| Digital and academic literacies |
| Quantitative and qualitative research |
| Policy and funding allocation resources |
| Ethical conduct of global health practice and research |
| Trends and determinants of global disease patterns |
| Cultural competency |
| Global health governance and diplomacy |
| Project management |
| Ethics and human rights |
| Epidemiology of tropical diseases |
| Population mobility |
| Social studies (culture, social responsibility, humanism, and social change) |
| Socio-economic determinants of health |
| Health care services |
| Health systems |
| Global governance |
| Environmental change |
| Population health |
| Globalisation |
| Human rights and ethics |
| International development |
| Monitoring and evaluation |
| Management and leadership |
| Policy analysis and development |
| Impact of globalisation on population health, health systems and healthcare |
| Major global health initiatives and efforts |
| Determinants of health |
| Ethics, health equity and social justice |
| Sociocultural, political awareness and policy promotion |
| Collaboration, partnering and communication |
| Capacity strengthening |
| Personal competencies and professional practice |
| Global health domain |
| Geography |
| Transborder |
| North/South |
| National reference |
| Regional reference |
| Local |
| Cooperation |
| Intergovernmental organisation reference |
| Non-governmental organisation reference |
| Government reference |
| Interdependence/otherness |
| Individual/population |
| Illness prevention |
| Health promotion |
| Primary health care |
| Clinical care |
| Health equity trade |
| Foreign direct investment |
| Aid |
| Gender |
| Global health ethics |
| Medicine |
| Sociology/anthropology |
| Epidemiology |
| Public health |
| Political science |
| History |
| Philosophy |
| Law |
| Engineering |
| Postcolonialism |
| Neoliberalism |
| Equity |
| Health determinants |
| Environmental degradation |
| Socioeconomic status issues |
| Food |
| Trade |
| Governance |
| War/disaster |
| Applied research |
| Interdisciplinarity |
| Health disparities |
| Original domains: |
| Global burden of disease and trends |
| Determinants of health |
| Environmental health |
| Health systems and health/nursing care delivery |
| Health policy, economics and global health governance |
| Sociocultural and ethical factors in health and healthcare |
| Global health competencies: |
| Global burden of disease and trends in morbidities and mortalities |
| Healthcare disparities between countries and determinants of health |
| Demographic and epidemiological transitions (global ageing and urbanization) |
| Cooperation in healthcare, foreign policy and diplomacy, and their force in health, healthcare and social services |
| Structure and function of health systems in different parts of the world |
| Working as international health experts |
| Cultural expertise and intercultural communication |
| MDG’s, poverty reduction and gender equality |
| Primary health care in different parts of the world |
| Epidemiological methods and global health research |
| Globalization of health and healthcare issues |
| Environmental factors |
| Social and economic determinants of health |
| Health care systems |
| Global health policy and governance |
| Human rights |
| Collaborating and partnering |
| Ethical reasoning and professional practice |
| Program management |
| Global health systems |
| Social, economic and environmental determinants of health |
| Global health challenges, issues and trends |
| Global health status and disparities |
| Ethics, social justice, and human rights |
| Cultural influences on health and healthcare |
| Global health research |
| Global health competencies |
| Global burden of disease/ trends in global health |
| Social and environmental determinants of health |
| Health care in low resources settings |
| Global health ethic |
| Globalization of health care |
| Health care system |
| Primary health care |
| Health equity |
| Collaboration and networking in global health |
| Disease prevention and health promotion |
| International development and cooperation |
| Global health leadership |
| Global health research |
| Ethics, human rights and equity |
| Cultural awareness and sensitivity |
| Gender and global health |
| An overview of global health |
| Global burden of disease and injury by region, country, gender, and age |
| Determinants of individual and population health and health inequalities |
| Human rights, ethics, and development |
| Global health governance, health systems and health financing |
| Major global health initiatives |
| Main actors in global health: public, private and civil society |
| Communication and advocacy |
| Program and project management |
| Public health, including policy, demography, health systems, determinants of health and epidemiology |
| Economics, including macroeconomics and microeconomics |
| Statistics, biostatistics and epidemiology |
| Medicine/nursing |
| Ethics |
| Law and human rights |
| Area specific inside the global health frameworks |
| Case study methodology |
| Optional area-specific module |
| Sociocultural fieldwork |
| Advocating effectively for the improvement of health systems and health equity |
| Demonstrating critical consciousness of issues that affect the health of people around the world |
| Key global health domains, principles and competencies |
| The global burden of disease and its determinants |
| Global epidemiology |
| The human rights dimensions of global health problems |
| Global health governance |
| Health systems and health professionals |
| Diversity, human rights and ethics |
| Environmental, social and economic determinants of health |
| Global epidemiology |
| Global health governance |
| Health systems and health professionals |
| Assist students to manage patients with diseases and clinical presentations common to voyagers and their hosts |
| Global health theory |
| Primary care within diverse cultural settings |
| Health-care disparity between the host and the visitor’s usual settings |
| Immigrant health |
| Role of primary care in global health |
| Role of colleagues in other disciplines in global health |
| Global health clerkship recommendations: |
| Health implications of travel, migration and displacement |
| Social and economic determinants of health |
| Population, resources and the environment |
| Healthcare in low resource settings |
| Human rights in global health |
| Health best practices and health systems |
| Fulfilling professional and ethical obligations as a global citizen |
| Examine the role of history, politics, culture, gender, religion, environment and socioeconomic status in increasing vulnerability and poor health throughout the world |
| Analyse the major health challenges facing the world from a multidisciplinary perspective |
| Analyse the factors that influence public health in resource-poor settings |
| Examine various effective interventions in tackling major health problems in resource-poor settings |
| Participate actively within a multidisciplinary team in tackling health problems prevalent in resource-poor settings in the world |
| Role of major Canadian and international health organizations |
| Approach to the management and assessment of health issues |
| Recognize that sustainable multidisciplinary development requires partnership at the community level |
| Key political and economic stakeholders, policies and programs that shape the social determinants of health |
| Advantages and challenges of different models of healthcare delivery |
| Identify vulnerable or marginalized population |
| Role of primary health care |
| Utilize population and disease surveillance databases |
| Ethical principles of clinical and translational research |
| Ethical challenges |
| Interact respectfully with other cultures |
| Assess the potential of the clinician’s skills and societal position |
| Potential for unintended consequences |
| Socioeconomic and environmental determinants of health |
| Cultural diversity and health |
| Health implications of migration, travel and displacement |
| Health as a human right and development resource |
| Globalization and health |
| International comparison of disease burden |
| International comparison of health systems |
| International elective and exchange opportunities |
| International health and development |
| International movement of people |
| Tropical medicine |

Competencies by concept used in the literature

In the literature several terms where used to refer to global health education. 3 principal terms were found: Global Health, Global Public Health, Global Medical Education. In a first instance, the competencies were organised in three sub-tables by the term used in the literature to refer to global health education.

| **Global Health** | | | |
| --- | --- | --- | --- |
| **Level** | **Reference** | **Method** | **Competencies** |
| International | Consortium of University for Global Health | Established own competencies:  Two staged study to develop a global health competency framework in 2014 through a multi-phased consultation process that engaged a diverse panel of experts with interdisciplinary backgrounds. | - Global burden of disease - Globalization of health and healthcare - Social and environmental determinants of health - Capacity strengthening collaboration, partnering and communication - Ethics - Professional practice - Health equity and social justice - Programme management - Sociocultural and political awareness - Strategic analysis |
| International | Tiwari R, English R, Begg K, Chikte U. Re-envisioning Global Health Competencies for the African region aligned with local health needs and resources. Annals of Global Health. 2022;88(1). doi:10.5334/aogh.3844 | Established own competencies:  Developed a set of global health competencies for the African region. Comprising scan of competencies, literature review and expert discussion conducted 2017–2020. | Seven core:   - Global health systems and international relations - Global evidence ecosystem - Role of international organizations - Universal health issues - Intellectual property rights - Responses to issues affecting different at-risk groups - Local, national, and international policy and economic context affecting global health   Four cross-cutting:   - Digital and academic literacies - Quantitative and qualitative research - Policy and funding allocation resources - Ethical conduct of global health practice and research |
| International | Association of Pacific Rim Universities  Withers M, Lin H-H, Schmidt T, delos Trinos J, Kumar S. Establishing competencies for a global health workforce: Recommendations from the Association of Pacific Rim Universities. Annals of Global Health. 2019;85(1). doi:10.5334/aogh.32 | Established own competencies:  Convened a workshop with 30 faculty, university administrators, students, and NGO workers representing both the Global North and South to gain consensus on core competencies in masters’-level global health training | - Trends and determinants of global disease patterns - Cultural competency - Global health governance and diplomacy - Project management - Ethics and human rights |
| International | Harmer A, Lee K, Petty N. Global Health Education in the United Kingdom: A Review of University undergraduate and postgraduate programmes and courses. Public Health. 2015;129(6):797–809. doi:10.1016/j.puhe.2014.12.015 | Synthesised competencies from literature:  Systematic review of the literature to identify a set of global health core competencies. Then compared list of core competencies to global health programmes offered in the UK. | - Global burden of disease - Epidemiology of tropical diseases - Population mobility - Social studies (culture, social responsibility, humanism, and social change) - Socio-economic determinants of health - Health care services - Health systems - Global governance - Environmental change - Population health - Globalisation - Human rights and ethics - International development - Monitoring and evaluation - Management and leadership - Policy analysis and development |
| China | Ding W, Guan Y, Peterhans B, Hoffmann A, Zhou X-N. Adaptation of the CUGH global health competency framework in the Chinese context: A mixed-methods study. Global Health Research and Policy. 2023 Nov 2;8(1). doi:10.1186/s41256-023-00327-w | Established own competencies:  Conducted a two stage study. The first stage aimed to adapt the CUGH global health competency framework to the Chinese context using a modified Delphi consultation approach involving a panel of global health experts in China. The second stage focused on prioritizing the adapted competencies and gathering insights for the enhancement of educational programs catering to Chinese public health professionals. | ‘What’ of global health – Establishing what is global health:   - Global burden of disease - Impact of globalisation on population health, health systems and healthcare - Major global health intiatives and efforts   ‘Why’ of global health – Ensuring awareness of different socio-cultural, political and environmental settings:   - Determinats of health - Ethics, health equity and social justice - Sociocultural, poltical awareness and policy promotion   ‘How’ of global health – Ability to review global health problems, develop an approach to address, identify monitoring and evaluation:   - Collaboration, partnering and communication - Programme management - Capacity strengthening - Personal competencies and professional practice |
| North America  (Unites States and Canada) | Lencucha R, Mohindra K. A snapshot of Global Health Education at North American Universities. Global Health Promotion. 2014;21(1):63–7. doi:10.1177/1757975913514464 | Identified what is being taught:  Identified the content areas being covered in global health curricula in North American universities. Collected 67 course syllabi and analyzed the topics covered. | Geography   - Transborder - North/South - National reference - Regional reference - Local   Cooperation   - Intergovernmental organisation reference - Non-governmental organisation reference - Government reference - Interdependence/otherness   Individual/population   - Illness prevention - Health promotion - Primary health care - Clinical care   Health equity trade   - Foreign direct investment - Aid - Gender - Global health ethics   Discipline department   - Medicine - Sociology/anthropology - Epidemiology - Public health - Political science - History - Geography - Philosophy - Law - Engineering   Perspectives   - Postcolonialism - Gender - Neoliberalism - Equity   Health determinants   - Environmental degradation - Socioeconomic status issues - Food - Trade - Governance - War/disaster |
| North America  (United States and Canada) | Jacobsen K, Li X, Gartin M, Malouin R, Waggett C. Master of science (MS) and Master of Arts (MA) degrees in Global Health: Applying Interdisciplinary Research Skills to the study of globalization-related health disparities. Pedagogy in Health Promotion. 2020;6(1):14–22. doi:10.1177/2373379919895032 | Identified what is being taught:  Identified global health master degrees in North America. Curricula and syllabi were codified, grouped and then thematised. | 4 themes:   - Applied research - Interdisciplinarity - Health disparities - Globalisation |
| **Global Public Health** | | | |
| **Level** | **Reference** | **Method** | **Competencies** |
| International | Association of Schools and Programmes of Public Health (ASPPH) 2011  Association of Schools and Programs of Public Health [Internet]. 2011 [cited 2024 May 2]. Available from:  https://www.publichealth.  pitt.edu/Portals/0/Main  /ASPH%20GH%  20Competencies.pdf | ASPPH launched a comprehensive initiative (the Global Health core Competency Development) to identify core global health competencies expected of public health students. | 5 domains:   - Capacity strengthening - Collaborating and partnering - Ethical reasoning and professional practice - Health equity and social justice   Program management |
| International | Association of Schools and Programmes of Public Health (ASPPH)  MPH concentrations in Global Health 2018  Association of Schools and Programs of Public Health [Internet]. 2018 [cited 2024 May 2]. Available from: https://aspph-prod-  webassets.s3.amazonaws  .com/GH-competencies-Toolkit.pdf | Established own competencies:  The Council on Education for Public Health (CEPH)- accredited institutions are involved in teaching and/or planning curricula for Master of Public Health (MPH) students concentrating in global health.  The ASPPH Master of Public Health’s Global Health Concentration Competencies built on top of CEPH’s 2016 accreditation criteria. | 6 domains:   - Analyze the roles, relationships, and resources of the entities influencing global health - Apply ethical approaches in global health research and practice - Apply monitoring and evaluation techniques to global health programs, policies, and outcomes - Propose sustainable and evidence-based multi-sectoral interventions, considering the social determinants of health specific to the local area - Design sustainable workforce development strategies for resource- limited settings   Display critical self- reflection, cultural humility, and ongoing learning in global health |
| **Global Medical Education** | | | |
| **Level** | **Reference** | **Method** | **Competencies** |
| International | Walpole S, Shortall C, van Schalkwyk M, Merriel A, Ellis J, Obolensky L, et al. Time to go global: A Consultation on Global Health Competencies for postgraduate doctors. International Health. 2016;8(5):317–23. doi:10.1093/inthealth/ihw019 | Established own competencies:  Conducted a literature review to develop global health competencies. List of competencies was reviewed by modified Delphi consultation with stakeholders worldwide. | - Diversity, human rights and ethics - Environmental, social and economic determinants of health - Global epidemiology - Global health governance   Health systems and health professionals |
| International | Battat R, Seidman G, Chadi N, Chanda M, Nehme J, Hulme J, et al. Global Health Competencies and approaches in medical education: A literature review. BMC Medical Education. 2010 Dec;10(1). doi:10.1186/1472-6920-10-94 | Synthesised competencies from literature:  Conducted a literature review to identify competencies and educational approaches for teaching global health in medical schools | - Global burden of disease - Travel medicine - Healthcare disparities between countries - Immigrant health - Primary care within diverse cultural settings   Skills to better interface with different populations, cultures and healthcare systems |
| North America  (United States and Canada) | Global Health Education Consortium (GHEC) and the Association of Faculties of Medicine (AFMC) Joint Committee  Arthur M, Battat R, Brewer T. Teaching the basics: Core competencies in global health. Infectious Disease Clinics of North America. 2011 Jun;25(2):347–58. doi:10.1016/j.idc.2011.02.013 | Established own competencies:  Conducted a literature review to develop a list of core competencies in global health for general medical education. List of competencies was peer reviewed using a modified Delphi method. | - Global burden of disease - Health implications of travel, migration and displacement - Social and economic determinants of health - Population, resources and the environment - Globalisation of health and healthcare - Healthcare in low-resource settings   Human rights in global health |
| Canada | Canadian Federation of Medical Students, Canada | Established own competencies:  Adapted GHEC/AFMC competencies to a Canadian which was then peer reviewed by global health education leaders across North America. | Medical expert:   - Global burden of disease - Role of major Canadian and international health organizations - Approach to the management and assessment of health issues   Communicator:   - Cultural competency   Collaborator:   - Recognize that sustainable multidisciplinary development requires partnership at the community level   Leader:   - Key political and economic stakeholders, policies and programs that shape the social determinants of health - Advantages and challenges of different models of healthcare delivery   Health advocate:   - Determinants of health - Identify vulnerable or marginalized population - Role of primary health care   Scholar:   - Utilize population and disease surveillance databases - Ethical principles of clinical and translational research   Professional:   - Ethical challenges - Interact respectfully with other cultures - Assess the potential of the clinician’s skills and societal position   Potential for unintended consequences |
| United Kingdom | Johnson O, Bailey S, Willott C, Crocker-Buque T, Jessop V, Birch M, et al. Global Health Learning Outcomes for medical students in the UK. The Lancet. 2012 Jun;379(9831):2033–5. doi:10.1016/s0140-6736(11)61582-1 | Established own competencies:  The Global Health Learning Outcomes Working Group developed a list of recommended global health competencies for UK medical students. These were extrapolated from the mandatory undergraduate learning outcomes listed in the General Medical Council's report Tomorrow's Doctors. | - Global burden of disease - Socioeconomic and environmental determinants of health - Health systems - Global health governance - Human rights and ethics   Cultural diversity and health |
| United States,  Canada,  Latin America,  Caribbean | Wilson L, Harper D, Tami-Maury I, Zarate R, Salas S, Farley J, et al. Global Health Competencies for Nurses in the Americas. Journal of Professional Nursing. 2012;28(4):213–22. doi:10.1016/j.profnurs.2011.11.021 | Survey to nurses in Americas to identify their perceptions of global health competencies for undergraduate nursing students. Survey included questions based on GHEC/AFMC competencies. |  |
| Brazil | Ventura et al., (2014), Brazil | Created questionnaire on global health competencies sent to Brazilian nurses. Questionaire included 30 competencies, divided in six domains. | Six domains:   - Global burden of disease - Health implications of migration, travel and displacement - Social and environmental determinants of health - Globalization of health and healthcare - Healthcare in low-resource settings   Health as a human right and development resource |
| International | Rowson M, Smith A, Hughes R, Johnson O, Maini A, Martin S, et al. The evolution of Global Health Teaching in undergraduate medical curricula. Globalization and Health. 2012;8(1):35. doi:10.1186/1744-8603-8-35 | Conducted survey study sent to medical schools across the world in an effort to analyse their teaching of global health.  Developed topics included in survey based on authors own knowledge. | Survey topic checklist:   - Globalization and health - International comparison of disease burden - International comparison of health systems - International elective and exchange opportunities - International health and development - International movement of people - Travel medicine   Tropical medicine |
