# Supplementary figures and images for: Global health education programs: Are we embedding contemporary global health needs into the curriculum of master’s programs?
Source: Front Public Health. 2026 Jan 9;13:1697295. doi: 10.3389/fpubh.2025.1697295 (PMC12827718; doi:10.3389/fpubh.2025.1697295)

**Supplementary Material 3. Map of graduate global health programmes.**

**
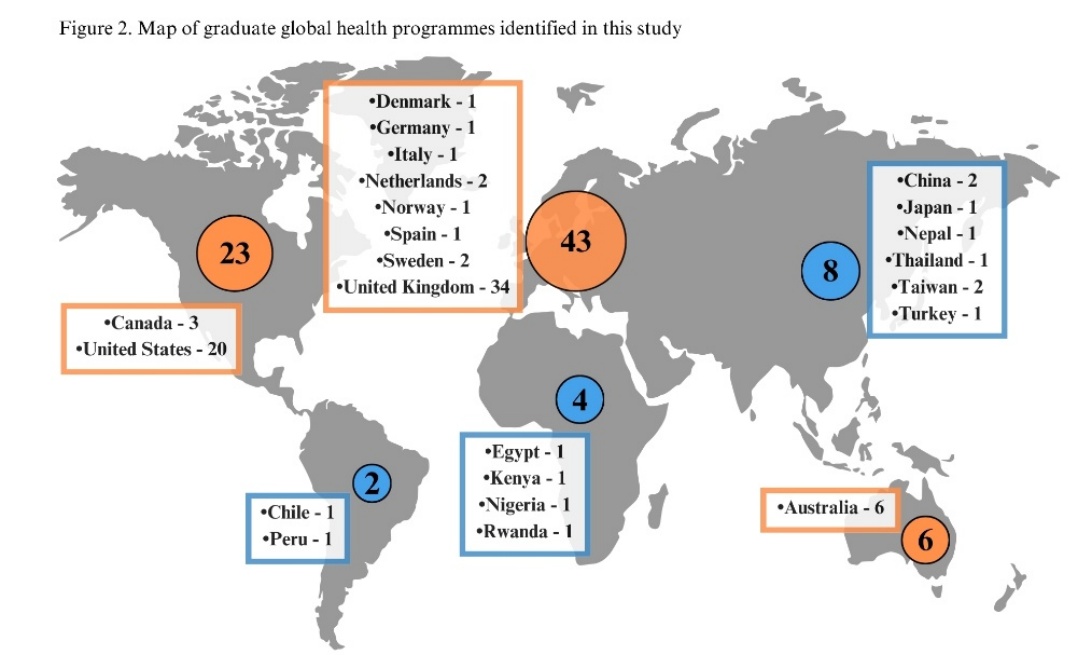
**

Supplement: Supplementary file 3 [file Table_3.docx]
