# Supplementary material for: Global health education programs: Are we embedding contemporary global health needs into the curriculum of master’s programs?
Source: Front Public Health. 2026 Jan 9;13:1697295. doi: 10.3389/fpubh.2025.1697295 (PMC12827718; doi:10.3389/fpubh.2025.1697295)
Supplement: Supplementary file 4 [file Table_4.docx]

**Supplementary Material 4. Module coverage variation across the Global North and South.**

| **Similar Coverage** | **Greater Coverage in North^1^** | **Greater Coverage in South** |
| --- | --- | --- |
| Infectious Diseases | Global Health Challenges | Health-Environmental-Sustainability |
| Health Systems and Governance | Thesis, Written Research | Biostatistics |
| Health Nutrition | *Bioscience* | Epidemiology |
| Health Information and Informatics | *Anthropology* | Social and Cultural Context of Health |
| Foundations of Global Health | *Bioterrorism and Health Intelligence* | Law, Ethics and Rights |
| Research Methods | *Digital Health* | Public Health |
| Humanitarianism and Disaster Management | *Human Geography and Health* | Politics and Policy |
| International Development and Health | *Molecular Biology* | Global Health Professional Skills |
| Migrant Health |  | Leadership and Management |
| Health Promotion Design, Monitoring, Evaluation |  | *Human Security and Global Health* |
| Health Economics |  | *Occupational Health* |
| Advocacy and Justice |  | *Travel Medicine* |
| Gender, Sexuality, Reproductive and Child Health |  |  |

**Legend:** *Italic* modules are solely found in that specified geographic location.

^1^Greater coverage was defined as the difference of more than 10% in module coverage between northern and southern institutions.
